# Quantum-circuit black hole lasers

**DOI:** 10.1038/s41598-021-98456-0

**Published:** 2021-09-27

**Authors:** Haruna Katayama

**Affiliations:** Graduate School of Advanced Science and Engineering, Hiroshima University, Higashihiroshima, 739-8521 Japan

**Keywords:** General relativity and gravity, Quantum simulation

## Abstract

A black hole laser in analogues of gravity amplifies Hawking radiation, which is unlikely to be measured in real black holes, and makes it observable. There have been proposals to realize such black hole lasers in various systems. However, no progress has been made in *electric circuits* for a long time, despite their many advantages such as high-precision electromagnetic wave detection. Here we propose a black hole laser in Josephson transmission lines incorporating metamaterial elements capable of producing Hawking-pair propagation modes and a Kerr nonlinearity due to the Josephson nonlinear inductance. A single dark soliton obeying the nonlinear Schrödinger equation produces a black hole-white hole horizon pair that acts as a laser cavity through a change in the refractive index due to the Kerr effect. We show that the resulting laser is a squeezed-state laser characterized by squeezing parameters. We also evaluate the degree of quantum correlation between Hawking and its partner radiations using entanglement entropy which does not require simultaneous measurements between them. As a result, the obtained entanglement entropy depending on the soliton velocity provides strong evidence that the resulting laser is derived from Hawking radiation with quantum correlation generated by pair production from the vacuum.

## Introduction

There has been considerable effort to construct “The Theory of Everything” that unifies the four fundamental forces. The last remaining urgent issue is to integrate general relativity and quantum mechanics. One of the rare phenomena they encounter is Hawking radiation^1^ from a black hole where even light cannot escape. Hawking radiation is the particle emission caused by quantum-mechanical pair production near the event horizon. Therefore, the observation of the Hawking radiation is a key for integrating general relativity and quantum mechanics. However, Hawking radiation is unlikely to be measured from a real black hole because it is much smaller than the background radiation.

The idea of black hole analogues in laboratory systems came up to study Hawking radiation instead of that emitted from an actual black hole. They can be realized by designing a system in which a reference wave cannot escape from a background flow with a spatially varying velocity. The velocities of the background flow and the reference wave play the roles of the free-fall velocity and electromagnetic wave velocity in an actual black hole. Since Unruh^2^ opened up the study in sonic systems based on the idea, analogue black holes have been proposed in various systems such as liquid helium^3^, optical fibers^4^, Bose-Einstein condensates^5^, and electric circuits^6–11^. Later, an extremely unique proposal was put forward by Corley and Jacobson^12–14^ to further enhance the Hawking radiation called the black hole laser, which amplifies Hawking radiation by stimulated emissions in the analogue cavities consisting of both a black hole and a white hole for a reliable observation. They showed that for the bosonic field, the negative energy partner goes back and forth between two horizons if the dispersion is superluminal. Some of the particles with negative energy are transformed into particles with positive energy by the mode conversion caused on the event horizons during repeating these processes. It leads to an amplification of the Hawking process.

Therefore, the black hole laser allows us to make Hawking radiation observable. In fact, experimental studies on black hole lasers have progressed in BECs^15^. In contrast, despite significant advances in detection techniques on extremely weak electromagnetic fields in the study of the dynamical Casimir effect^16^, there have been no reports on black hole lasers in electric circuits for more than a decade since the seminal work on Hawking radiation by Schützhold and Unruh^6^ and the subsequent detailed study by Nation et al.^7^. This might be due to the absence of the anomalous dispersion required for black hole lasers in the previous electric circuits. In addition, nonlinear effects have not been actively considered because they are not a necessary condition for analogue gravity effects.

Here, we propose black hole lasers in Josephson transmission lines by introducing *metamaterial* elements into the circuit that realize particle-antiparticle pair propagation modes possible with anomalous dispersion into a normal one, as well as the Kerr nonlinearity for controlling propagating mode selection in the cavity. This proposal is equivalent to creating the same situation in a circuit as an optical black hole laser using an optical fiber^17^. In addition, we evaluate the entanglement entropy, which measures the degree of entanglement of particles and antiparticles created in pair production near the event horizons, in order to confirm that the emitted light is surely Hawking radiation. It is revealed that entanglement entropy is characterized by squeezing parameters related to Hawking temperatures, which depend on the velocity of the soliton.

## Results

### Model

The black hole laser originally requires the superluminal dispersion with a positive curvature in the dispersion curve of the system^12–14^. Here we use a model in which a black hole laser is feasible even in subluminal dispersion with well-designed dispersion curves^17^. To create the dispersion relation required for black hole lasers in subluminal dispersion, we employ dispersive engineering utilizing *metamaterials* made of sub-wavelength inclusions that provide tremendous degrees of freedom for manipulating with high precision the electromagnetic parameters of materials and modes. In fact, metamaterials create a medium in which the permittivity and permeability are simultaneously negative, which does not exist in nature, and enables the unique property that the phase velocity and group velocity of electromagnetic waves are opposite to each other. In addition, the Josephson effect provides the Kerr nonlinearity^18, 19^ essential for black hole lasers, which determines the group velocity, required to select the propagation modes in the system.

**Figure 1:**
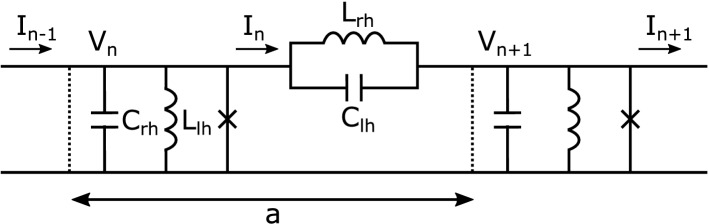
Schematic representation of the composite right/left-handed nonlinear transmission line. Each unit cell consists of the series branch elements and the shunt branch elements. In the series branch, a linear inductive element of inductance $L_{rh}$ is arranged in parallel with a linear capacitance $C_{lh}$. These constitute the linear dispersive element of the line. While in the shunt branch, a linear inductive element of inductance $L_{lh}$ is also arranged in parallel with a linear capacitor of capacitance $C_{rh}$ as well as the Josephson element (represented by $\times$) which is responsible for the nonlinearity of the system. The dotted vertical lines mark the unit cell of the lattice with the length *a*.

Suppose that a Josephson *metamaterial* transmission line consists of a number of LC blocks each comprised of composite right/left-handed components together with a Josephson element in the shunt branch as illustrated in Fig. 1. Starting from the application of Kirchhoff’s law to this system together with the Josephson relation, the current conservation at the *n*th node is expressed as

1 $$\begin{aligned} I_{n}-I_{n-1}=-I_{J, n}-I_{C_{r h}, n}-I_{L_{lh},n}, \end{aligned}$$

where $I_n$ is the current through the *n*th node comprising of the current through the right-handed (rh) inductor with inductance $L_{rh}$ and the left-handed (lh) capacitor with capacitance $C_{lh}$ at the *n*th cell, i.e., $I_{n}=I_{L_{rh},n}+I_{C_{lh},n},$

2 $$\begin{aligned} I_{L_{rh}, n}&=-\frac{\hbar }{2 e} \frac{1}{L_{r h}}\left( \theta _{n+1}-\theta _{n}\right) , \end{aligned}$$

3 $$\begin{aligned} I_{C_{lh}, n}&=-\frac{\hbar }{2 e} C_{lh} \frac{d^{2}}{d t^{2}}\left( \theta _{n+1}-\theta _{n}\right) , \end{aligned}$$

where $I_{c}$, $\hbar$, *e*, and $\theta _n$ are the Josephson critical current, Dirac’s constant, an elementary charge, and the phase difference in the *n*th junction, respectively. The currents on the right-hand side of Eq. (1) are the Josephson current $I_{J, n}$, the displacement current $I_{C_{r h},n}$ flowing through the *n*th Josephson junction with capacitance $C_{rh}$, and the current $I_{L_{lh},n}$ through the left-handed inductor with inductance $L_{lh}$. Combining these relations, we obtain the following circuit equation,

4 $$\begin{aligned} C_{rh} \frac{d^{2} \theta _{n}}{d t^2}+\frac{1}{L_{l h}}\theta _{n}+ \frac{1}{L_J} \left( \theta _{n}-\frac{\theta _{n}^{3}}{3 !}\right) -\left( \frac{1}{L_{rh}}+C_{lh} \frac{d^{2}}{d t^{2}}\right) \left( \theta _{n+1}+\theta _{n-1}-2 \theta _{n}\right) =0, \end{aligned}$$

where we use $\sin \theta _n\simeq \theta _n-\theta _n^3/6$ and $L_{J}=\hbar /(2 e I_{c})$.

Now let us derive the dispersion relation of this transmission line by ignoring the nonlinear terms of the Josephson effect. We substitute a plane-wave solution $\theta _n\sim \exp [i(kna-\omega t)]$ with the wavenumber *k*, the frequency $\omega$, and unit cell length *a* for Eq. (4) and obtain the dispersion relation

5 $$\begin{aligned} \sin ^2 \frac{ka}{2}=\frac{1}{4}\left\{ \frac{\omega ^2}{\omega _{r h}^2}-L_{rh}\left( \frac{1}{L_{l h}}+ \frac{1}{L_{J}}\right) \right\} \left( 1-\gamma \frac{\omega ^{2}}{\omega _{r h}^{2}} \right) ^{-1}, \end{aligned}$$

where $\omega _{r h}=1/\sqrt{C_{r h} L_{r h}}$ and $\gamma =C_{l h}/C_{r h}$. In the regime of $\gamma \omega ^2\ll \omega _{rh}^2$, this reduces to

6 $$\begin{aligned} ka\simeq \frac{\omega }{\omega _{r h}}+\frac{\gamma }{2}\frac{\omega ^{3}}{\omega _{r h}^3}, \end{aligned}$$

by designing the circuit so that $L_{l h}=-L_J$^20^. This dispersion relation is the same as that of the optical fiber in which the black hole laser is considered^17^. Thus, our proposed circuit can be regarded as a circuit version of an optical fiber^17, 21^ at the nanometer scale.

**Figure 2:**
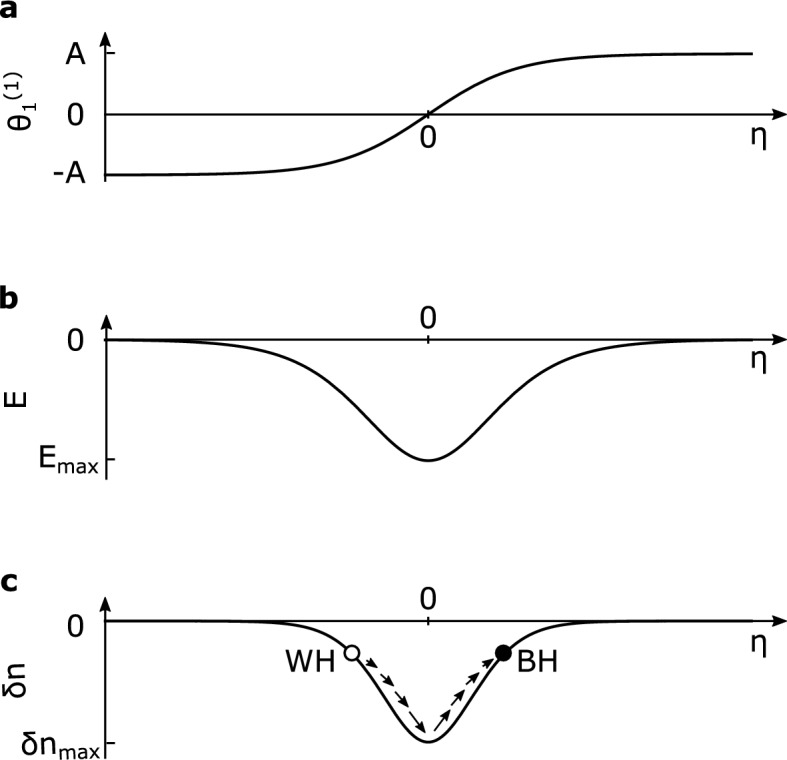
Sketch of (**a**) the phase soliton with the amplitude *A*, (**b**) the electric field with the amplitude $E_{max}=(-\hbar /2ed)A^2\sqrt{|Q/2P|}u$, and (**c**) the cavity formed by the *single* dark soliton with the amplitude $\delta n_{max}$. The filled circle and open circle represent the horizon of the black hole and white hole, respectively. The size of arrows representing the velocity of the probe pulse varies in space.

Our circuit equation contains a third-order nonlinear term just like an optical fiber, so the existence of a nonlinear wave is highly expected. Next, let us explore the waves hidden in the circuit equation (4) by using the discrete reductive perturbation method as follows^22–27^. We introduce two slow stretched space and time variables $\xi =\varepsilon \left( na-v_{g} t\right)$ and $\tau =\varepsilon ^{2} t$ with the small dimensionless parameter $\varepsilon$ $(0< \varepsilon \ll 1)$ and group velocity $v_g$ to separate fast and slow variations of $\theta _n$ and expand $\theta _n$ in principle as

7 $$\begin{aligned} \theta _{n}=\theta ^{(0)}+\sum _{l=-\infty }^{+\infty } \sum _{\alpha =1} \varepsilon ^{\alpha } \theta _{l}^{(\alpha )}(\xi ,\tau ) \exp [i l(k na-\omega t)]. \end{aligned}$$

Here, our analysis is restricted to the so-called rotating-wave approximation consisting essentially in neglecting higher harmonics,

8 $$\begin{aligned} \theta _{n}(t) \simeq \varepsilon \theta _{1}^{(1)}(\xi ,\tau )\exp [i(k na-\omega t)]+\varepsilon\theta _{1}^{*(1)}(\xi ,\tau )\exp [-i(k na-\omega t)]. \end{aligned}$$

Inserting these formulas into Eq. (4) in order to find balanced dispersion and nonlinearity yields the following equation for the $\varepsilon ^3$ order,

9 $$\begin{aligned} i \frac{\partial \theta _{1}^{(1)}}{\partial \tau }+P \frac{\partial ^{2} \theta _{1}^{(1)}}{\partial \xi ^{2}}+Q\left| \theta _{1}^{(1)}\right| ^{2} \theta _{1}^{(1)}=0, \end{aligned}$$

where

10 $$\begin{aligned} P&=\frac{\omega {\bar{\Omega }}a^2}{2} \left[ \cos k a-\omega _{c}^{2}\left( \frac{1}{\omega ^{2}}+\frac{3 \gamma }{\omega _{r h}^{2}}\right) \sin ^{2} ka\right] , \end{aligned}$$

11 $$\begin{aligned} Q&=\frac{1}{4\omega L_{J} C_c},\end{aligned}$$

12 $$\begin{aligned} C_c&=C_{rh}+4 C_{l h} \sin ^{2}\frac{ka}{2},\end{aligned}$$

13 $$\begin{aligned} {\bar{\Omega }}&=\frac{\omega _{c}^{2}}{\omega ^{2}}\left( 1-\gamma \frac{\omega ^{2}}{\omega _{r h}^{2}}\right) ,\end{aligned}$$

14 $$\begin{aligned} \omega _{c}&=\frac{1}{\sqrt{L_{rh} C_{c}}}. \end{aligned}$$

This is a well-known nonlinear Schrödinger equation found in various systems including optical fibers^28^, which contains soliton solutions. One such solution is a dark soliton expressed as,

15 $$\begin{aligned} \theta _{1}^{(1)}(\xi ,\tau )=A\,{\text {tanh}}\left( A \sqrt{\left| \frac{Q}{2 P}\right| }(\xi -u \tau )\right) e^{i(k\xi -\omega \tau)}, \end{aligned}$$

as shown in Fig. 2a, where *A* is the soliton amplitude and *u* is the relative soliton velocity in the $\xi -\tau$ coordinate. The solution holds under the condition $PQ <0$, which is always satisfied in our system.

### Black hole laser

Our system equipped with both the desired normal dispersion supporting pair-propagating modes and a Kerr effect is expected to be a circuit version of black hole lasers in optical fibers^17, 21^. Here we briefly review the black hole laser in an optical fiber as an example and reorganize the key parameters appropriate for our system. The fundamental idea is to amplify a probe pulse as Hawking radiation confined in the cavity formed by the two propagating solitons with the same velocity $v_s$ acting as mirrors in a conventional laser.

The event horizons occur at the points where the group velocity $v_{g}^{\mathrm {eff}}$ of the probe pulse in the system cannot keep up with the soliton velocity $v_s$, i.e., $v_g^{\mathrm {eff}}=v_s$. In other words, the probe pulse is trapped in the cavity and it cannot escape from the cavity, i.e., it cannot go outside the horizons classically. The effective group velocity under the Kerr modulations is given as

16 $$\begin{aligned} v_{g}^{\mathrm {eff}} =\frac{c}{n_{g}+\delta n(\eta )}, \end{aligned}$$

where $n_g$, $\delta n(\eta )$, and *c* represent the unperturbed group index, the refractive index perturbation modified by the Kerr effect of Josephson junctions in the comoving frame ($\eta =\xi -u \tau$), and the speed of light in vacuum, respectively.

**Figure 3:**
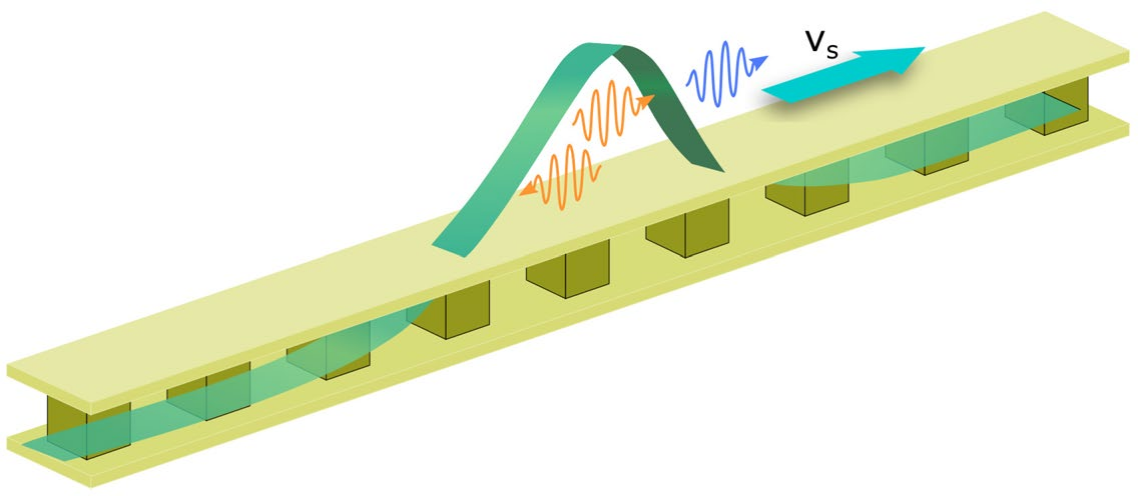
Schematic sketch of the black hole laser in the circuit. The refractive index perturbation $\delta n(\eta )$ (green) moving with the velocity $v_s$ changes the velocity of the probe pulses (orange wave packets) and classically traps them inside the soliton acting as the cavity. The probe pulses can be radiated quantum-mechanically as Hawking radiation (blue) by pair productions near the horizon.

The Kerr effect can cause a change in the refractive index depending on the strength of the electric field, equivalently the strength of the voltage in our circuit^29^. The refractive index perturbation under the soliton propagation is expressed as

17 $$\begin{aligned} \delta n(\eta )=\chi E^2, \end{aligned}$$

where $\chi$ is the third-order nonlinear susceptibility^30^, which is a negative constant in the case of the circuit with Josephson junctions^18, 19^ and the electric field *E* is given by *V*/*d* with *d* being the distance between plates. The voltage *V* across the junction is derived from the Josephson acceleration relation $V=(\hbar /2e)\partial \theta /\partial \tau$ and is expressed as

18 $$\begin{aligned} V=-\frac{\hbar }{2e}A^2\sqrt{\left| \frac{Q}{2P}\right| } \quad {\text {sech}}^2\left( A \sqrt{ \left| \frac{Q}{2P} \right| }\eta \right) . \end{aligned}$$

The soliton width *w* is roughly evaluated by $2\sqrt{|2P/Q|}/A$ and is about $w\simeq 150a$ for the soliton centered at the frequency $\omega _s=4.3 \times 10^9$ Hz with $A=0.01$, which is large enough to apply the continuum approximation. We plot the electric field *E* (Fig. 2b) and the refractive index perturbation $\delta n$ (Fig. 2c). The probe pulses are trapped in the soliton because the refractive index perturbation is negative^31^. In other words, a single soliton behaves as the cavity in our system as shown in Fig. 3.

**Figure 4:**
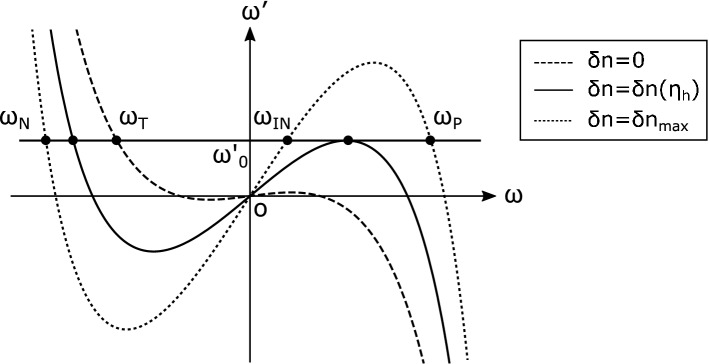
The relation between the frequencies in the laboratory frame $\omega$ and the comoving frame $\omega '$ by the Doppler shift. The dashed, solid, and dotted curved lines represent the relation at the perturbed index $\delta n=0$, $\delta n=\delta n(\eta _h)$, and $\delta n=\delta n_{max}$, respectively. The horizontal line shows the invariant frequency $\omega '_0$ in the comoving frame and the intersections with the curved lines correspond to the modes.

Now let us find the frequency modes satisfying the dispersion relation. The frequency $\omega '$ in the comoving frame is given by the Doppler relation as follows,

19 $$\begin{aligned} \omega '(\omega )=\omega -\omega \frac{v_s}{c}[n(\omega )+\delta n(\eta )], \end{aligned}$$

with the soliton velocity $v_s=u+v_g(\omega _s)$ in the laboratory frame. The modes are given by the solutions of $\omega '_0=\omega '(\omega )$, where $\omega '_0$ is the invariant frequency in the comoving frame for the given input frequency $\omega _{\mathrm {IN}}$. Figure 4 represents the Doppler relation in frequencies between the laboratory frame and comoving frame. The intersections of the horizontal line and the curved lines give the solutions. There are three modes $\omega _{\mathrm {IN}}$, $\omega _{\mathrm {P}}$, and $\omega _{\mathrm {N}}$ between horizons with $\delta n=\delta n_{max}$ and the other mode $\omega _{\mathrm {T}}$ exists outside the horizons with $\delta n=0$. The frequency $\omega _h$ at the event horizon satisfies $d\omega '/d\omega |_{\omega =\omega _h}=0$ and $\omega '(\omega _h)=\omega '_0$. We can find the position of the event horizon $\eta _{h}$ by solving these equations.

**Figure 5:**
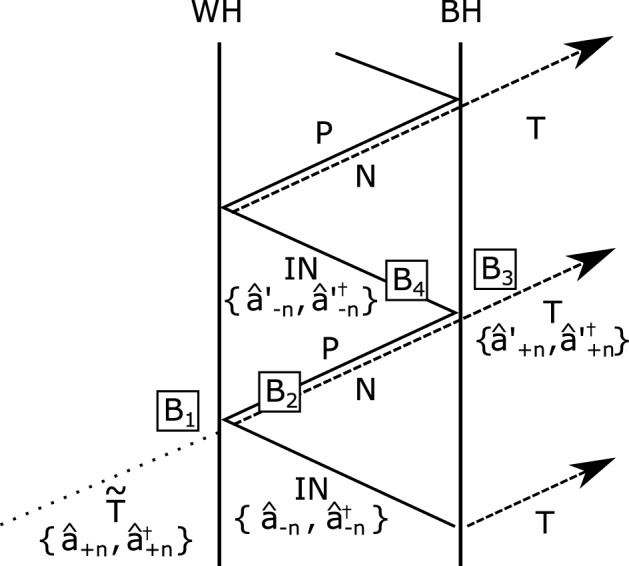
Sketch of the trajectories of modes. The solid (dashed) lines represent the mode with positive (negative) frequencies for antiparticles (particles). The dotted line is a virtual input mode for particles. The symbols are presented in the main text.

The evolution of these modes is shown in Fig. 5. The IN mode propagates towards the white hole horizon and turns back as the P mode to the black hole horizon together with the N mode where both modes undergo partial mode conversion. The P mode bounces at the black hole horizon and then becomes the IN mode propagating to the white hole horizon, while the N mode crosses the black hole horizon and is emitted as the T mode. The norm of the modes is conserved through the process as follows,

20 $$\begin{aligned} \Vert \mathrm {IN}_{n}\Vert =\Vert \mathrm {IN}_{n+1}\Vert +\Vert \mathrm {T}_{n+1}\Vert , \quad n \ge 0, \end{aligned}$$

where $\Vert \mathrm {X}_{n}\Vert$ denotes the norm defined by the Klein Gordon inner product of the *n*th X mode. Since $\Vert \mathrm {IN}_{n}\Vert >0$ and $\Vert \mathrm {T}_{n+1}\Vert <0$, this results in $\Vert \mathrm {IN}_{n+1}\Vert >\Vert \mathrm {IN}_{n}\Vert$. Therefore, the Hawking radiation is amplified by the mode transformations based on the norm conservation at the event horizon. This is the essential concept of the black hole laser^11–14^.

In addition, the nonlinear optical effect near the event horizons in our system becomes remarkable due to the presence of solitons. In the following, we incorporate the nonlinear mode conversions near the event horizon by adopting the model considered by Leonhardt et al.^32^ from the standpoint of nonlinear quantum optics and then find that our laser is a squeezed state laser. Consider the *n*th amplification process in the horizon cavity. The two modes pair-produced by mode conversion ($B_1$) at the white hole horizon propagate toward the black hole horizon ($B_2$). At the black hole horizon, the two modes again undergo a time-reversed mode conversion ($B_3$) opposite to the previous one and then propagate toward the departed white hole horizon ($B_4$). These four distinct processes are incorporated in the matrix *B*, allowing it to be expressed as

21 $$\begin{aligned} B=B_{4} B_{3} B_{2} B_{1}, \end{aligned}$$

where

22 $$\begin{aligned} B_{1}&=\left( \begin{array}{lr} \cosh \zeta &{} -\sinh \zeta \\ -\sinh \zeta &{} \cosh \zeta \end{array}\right) ,\end{aligned}$$

23 $$\begin{aligned} B_{2}&=\left( \begin{array}{cc} e^{-i \theta _{-}} &{} 0 \\ 0 &{} e^{i \theta _{+}} \end{array}\right) ,\end{aligned}$$

24 $$\begin{aligned} B_{3}&=\left( \begin{array}{cc} \cosh \zeta &{} \sinh \zeta \\ \sinh \zeta &{} \cosh \zeta \end{array}\right) ,\end{aligned}$$

25 $$\begin{aligned} B_{4}&=\left( \begin{array}{cc} e^{-i \theta _{0}} &{} 0 \\ 0 &{} 1 \end{array}\right) , \end{aligned}$$

with the phases $\theta _0$, $\theta _{-}$, and $\theta _{+}$ acquired during propagation for each mode and a squeezing parameter $\zeta$. The transfer matrix *B* essentially represents squeezing transformation. Therefore, the laser discussed here is nothing but a *squeezed state laser*. The squeezing parameter $\zeta$ is given by

26 $$\begin{aligned} \tanh ^{2} \zeta =e^{-\frac{\hbar \omega }{k_{B} T_H}}, \end{aligned}$$

where $k_B$ is the Boltzmann constant and $T_H$ is the Hawking temperature which is proportional to the gradient of the velocity as follows^8^,

27 $$\begin{aligned} T_{H}=\frac{\hbar }{2 \pi k_{B}}\left| \frac{\partial v_g^{\mathrm {eff}}}{\partial \eta }\right| _{\eta =\eta _{h}}, \end{aligned}$$

where

28 $$\begin{aligned} \left| \frac{\partial v_g^{\mathrm {eff}}}{\partial \eta }\right| _{\eta =\eta _{h}}= \frac{4A\delta n(\eta _h)}{c}\sqrt{\left| \frac{Q}{2 P}\right| }\tanh {\left( A\sqrt{\left| \frac{Q}{2 P}\right| }\eta _h\right) }u^2. \end{aligned}$$

**Figure 6:**
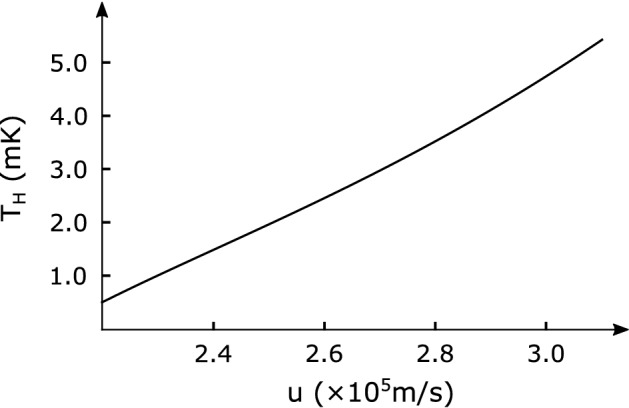
The dependence of the Hawking temperature $T_H$ on the relative soliton velocity *u*. We set the circuit parameters as $L_{rh}=4\times 10^{-8}\mathrm {H}$, $C_{rh}=2.5\times 10^{-16}\mathrm {F}$, $\gamma =5000$, $Ic =10^{-8}\mathrm {A}$, $\omega _0 = 2\times 10^8\mathrm {Hz}$, $\omega _s = 4.4 \times 10^9 \mathrm {Hz}$, and $a = 10^{-6}\mathrm {m}$.

The Hawking temperature depends on the relative soliton velocity *u* as depicted in Fig. 6. The Hawking temperature reaches the well-observable milli-Kelvin order under the circuit parameters feasible with current technology.

As a result, the input modes at the *n*th amplification process are transformed to the output modes by a Bogoliubov transformation *B* as follows,

29 $$\begin{aligned} \left( \begin{array}{l} {\hat{a}}_{-n}^{\prime } \\ {\hat{a}}_{+n}^{\prime \dagger } \end{array}\right) =B\left( \begin{array}{l} {\hat{a}}_{-n} \\ {\hat{a}}_{+n}^{\dagger } \end{array}\right) , \end{aligned}$$

where ${\hat{a}}_{+(-)n}$ $({\hat{a}}_{+(-)n}^{\dagger })$ represents annihilation (creation) operator of the *n*th input mode for particles (antiparticles), while ${\hat{a}}^{\prime }_{+(-)n}$ $({\hat{a}}_{+(-)n}^{\prime \dagger })$ are those of output modes for particles (antiparticles). The Bogoliubov transformation operator *B* is rewritten by

30 $$\begin{aligned} B=e^{i \psi }\left( \begin{array}{ll} \mu &{} \nu ^{*} \\ \nu &{} \mu ^{*} \end{array}\right) , \end{aligned}$$

where

31 $$\begin{aligned} \psi&=-\frac{1}{2}\left( \theta _{0}+\theta _{-}-\theta _{+}\right) , \end{aligned}$$

32 $$\begin{aligned} \mu&=e^{-\frac{i}{2}\left( \theta _{0}+\theta _{-}+\theta _{+}\right) } \cosh ^2 \zeta -e^{-\frac{i}{2}\left( \theta _{0}-\theta _{-}-\theta _{+}\right) } \sinh ^2\zeta ,\end{aligned}$$

33 $$\begin{aligned} \nu&=e^{\frac{i}{2}\left( \theta _{0}-\theta _{-}-\theta _{+}\right) } \sinh \zeta \cosh \zeta -e^{\frac{i}{2}\left( \theta _{0}+\theta _{-}+\theta _{+}\right) } \sinh \zeta \cosh \zeta , \end{aligned}$$

which satisfy with the conditions that $|\mu |^{2}-|\nu |^{2}=1$ and $\psi$ is real. The output modes serve as further input modes $({\hat{a}}^{\prime }_{-n}={\hat{a}}_{-(n+1)})$. Note that the frequency of Hawking radiation is negative in our system, as in the case with an optical fiber under normal dispersion. As shown in Fig. 5, the virtual particle in the $\tilde{\mathrm {T}}$ mode incident from the left side of the white hole event horizon and the antiparticle in the IN mode inside the cavity are input modes, while the particle in the outgoing T mode and the antiparticle in the IN mode are output modes.

**Figure 7:**
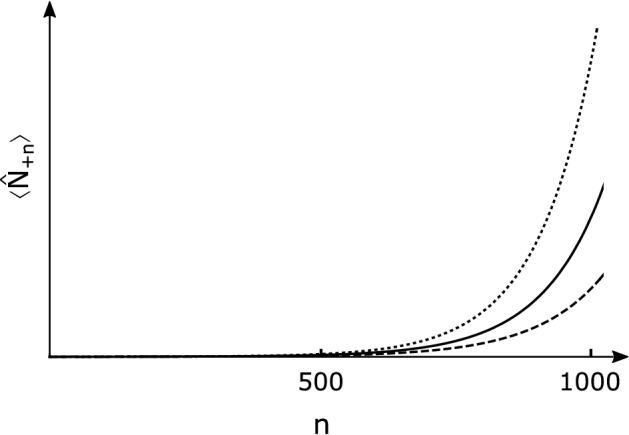
The number of outgoing particles as a function of the amplification steps at the fixed relative soliton velocities $u=2.99\times 10^5$m/s (dotted line), $u=3.00\times 10^5$m/s (solid line), and $u=3.01\times 10^5$m/s (dashed line). The circuit parameters are the same as in Fig. 6.

The number of particles outside the horizon after *n* steps amplified by our black hole laser is estimated as

34 $$\begin{aligned} \left\langle {\hat{N}}_{+n}\right\rangle&=\left\langle {\hat{a}}_{+n}^{\prime \dagger } {\hat{a}}_{+n}^{\prime }\right\rangle \nonumber \\&=|\mu |^{2 n}\left( 1-|\mu |^{-2}\right) , \end{aligned}$$

with

35 $$\begin{aligned} |\mu |^{2}=\frac{1}{2}\left[ 1+\cosh ^2 (2 \zeta )-\cos \left( \theta _{+}+\theta _{-}\right) \sinh ^2(2 \zeta ) \right] , \end{aligned}$$

where $\langle \cdots \rangle$ denotes the quantum-mechanical expectation. The amplification of the number of Hawking particles with each bounce process at the black hole horizon is depicted in Fig. 7 for the resonant case with $\cos \left( \theta _{+}+\theta _{-}\right) =0$. This typical enhancement shows surely lasing. Therefore, the resulting Hawking radiation is a squeezed state laser with squeezing parameters due to the nonlinearity of solitons.

**Figure 8:**
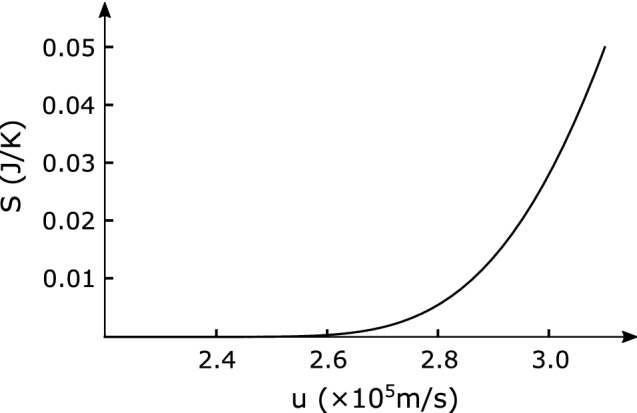
The dependence of the entanglement entropy *S* on the relative soliton velocity *u*. The circuit parameters are the same as in Fig. 6.

### Entanglement entropy

Hawking radiation originates from pair production from the vacuum inside near the horizon. The produced particle and antiparticle are inherently entangled with each other. Therefore, the detection of this entanglement is indispensable for the confirmation of Hawking radiation. However, it is unlikely to confirm the entanglement by conventional detection methods utilizing simultaneous observation of particle-antiparticle pairs because of the difficulty of detecting partner antiparticles left inside the horizon cavity. Here, we evaluate it using entanglement entropy thanks to the fact that an entangled particle bears the shadow of its partner particle. Entanglement entropy is a measure of quantum correlation between two particles labeled *A* and *B* and is defined by $S\left( \rho _{A}\right) =-{\text {Tr}}\left[ \rho _{A} \log \rho _{A}\right]$ where $\rho _{A}={\text {Tr}}_{B}\left( \rho _{A B}\right)$ is the reduced density matrix of a pure state density matrix $\rho _{A B}$. Therefore, the degree of entanglement can be evaluated without simultaneous observation of the partner particles.

Fortunately, our black hole laser is a laser with a two-mode squeezed state due to Hawking radiation and its partner. The quantum correlation between the two modes is naturally incorporated into the squeezed parameter. This squeezing parameter is responsible for the quantum correlation with unobservable partner particles trapped in the cavity. The entanglement entropy for the two-mode squeezed states is given in the well-known form^33, 34^ as

36 $$\begin{aligned} S=2 k_{B}\left[ \cosh ^{2} \zeta \ln \left( \cosh ^{2} \zeta \right) -\sinh ^{2} \zeta \ln \left( \sinh ^{2} \zeta \right) \right] . \end{aligned}$$

Note that the entanglement entropy depends on the relative soliton velocity *u* as shown in Fig. 8 since the squeezing parameter $\zeta$ involves the Hawking temperature depending on the soliton velocity. This soliton velocity dependence provides useful evidence for identifying the detected laser as being derived from Hawking radiation.

## Discussion

The black hole laser is an analogue gravity-derived laser that amplifies Hawking photons generated from vacuum fluctuations inside near the event horizon in a cavity formed by two horizons viewed as mirrors. It requires Hawking-related propagation modes with positive (particle) and negative (antiparticle) frequencies, which can be generated by using anomalous dispersion for example, in an analogue resonator. In this paper, we have applied dispersion engineering by adding metamaterial elements to ordinary transmission lines and deforming the dispersion relation by the Doppler effect used in optical fibers and achieved Hawking-related modes despite the ordinary dispersion relation in the transmission line. In addition, the third-order Kerr effect through the Josephson nonlinear inductance is introduced to control those modes. Based on these, we have proposed an *optical analogue* black hole laser in a Josephson transmission line with metamaterial elements. Unlike previous optical black hole lasers, our black hole laser still has a black hole/white hole cavity formed within a *single dark* soliton, where Hawking radiation is emitted into the normal region outside of solitons rather than inside of solitons. This selection can be achieved by controlling the Kerr effect through Josephson nonlinear inductance. Unfortunately, Hawking radiation has a negative frequency due to normal dispersion as with optical black hole lasers and differs from actual Hawking radiation with positive frequency. This will be solved if anomalous dispersion can be introduced in the transmission line.

We have also shown that our laser is a squeezed state laser based on Leonhardt’s quantum optical treatment of mode conversions in the horizon. This facilitates the analysis of the quantum entanglement required to identify the origin of Hawking radiation. In particular, entanglement entropy, which measures the degree of entanglement, is very effective when direct observation of partner radiation is difficult. The resulting entanglement entropy was found to be characterized by a squeezed parameter defined by the Hawking temperature that depends on the soliton velocity. It can be proved that Hawking radiation is strongly related to the soliton providing the analogue horizon if the entanglement entropy involved in this soliton velocity can be evaluated.

Black hole lasers have not been discussed in superconducting transmission lines so far. However, there are some advantages to the study of black hole lasers due to the latest technology accumulated in research such as quantum computers. In particular, the scalability and controllability of the system is an advantage over other systems. In addition, the detection of microwave photons and their quantum correlations in Josephson transmission lines has been proven through the study of the dynamical Casimir effect. The observation of Hawking radiation is also highly promising if our proposal is implemented.

## Data Availability

The data that support the findings of this study are available from the corresponding author upon request.
